# Flavor Profile Evolution of Bottle Aged Rosé and White Wines Sealed with Different Closures

**DOI:** 10.3390/molecules24050836

**Published:** 2019-02-27

**Authors:** Meng-Qi Ling, Han Xie, Yu-Bo Hua, Jian Cai, Si-Yu Li, Yi-Bin Lan, Ruo-Nan Li, Chang-Qing Duan, Ying Shi

**Affiliations:** 1 Center for Viticulture & Enology, College of Food Science and Nutritional Engineering, China Agricultural University, Beijing 100083, China; knighhtt@cau.edu.cn (M.-Q.L.); m18800135515@163.com (H.X.); siyuli@cau.edu.cn (S.-Y.L.); clanyibin@gmail.com (Y.-B.L.); liruonan0011@163.com (R.-N.L.); chqduan@cau.edu.cn (C.-Q.D.); 2 Key Laboratory of Viticulture and Enology, Ministry of Agriculture and Rural Affairs, Beijing 100083, China; 3 Shandong Taila Winery Co., Ltd., Weihai 264500, China; huayubo-111@163.com; 4 College of Biological Resources and Food Engineering, Qujing Normal University, Qujing 655011, China; caijian928@outlook.com

**Keywords:** rosé wines, white wines, bottle aging, flavor profile, closures

## Abstract

Bottle aging is the final stage before wines are drunk, and is considered as a maturation time when many chemical changes occur. To get a better understanding of the evolution of wines’ flavor profile, the flavor compounds (phenolic and volatile compounds), dissolved oxygen (DO), and flavor characters (OAVs and chromatic parameters) of rosé and dry white wines bottled with different closures were determined after 18 months’ bottle aging. The results showed the main phenolic change trends of rosé wines were decreasing while the trends of white wines were increasing, which could be the reason for their unique DO changing behaviors. Volatile compounds could be clustered into fluctuating, increasing, and decreasing groups using k-means algorithm. Most volatile compounds, especially some long-chain aliphatic acid esters (octanoates and decanoates), exhibited a lower decrease rate in rosé wines sealed with natural corks and white wines with screw caps. After 18 months of bottle aging, wines treated with natural corks and their alternatives could be distinguished into two groups based on flavor compounds via PLS-DA. As for flavor characters, the total intensity of aroma declined obviously compared with their initial counterparts. Rosé wines exhibit visual difference in color, whereas such a phenomenon was not observed in white wines.

## 1. Introduction

Wine flavor is composed of a wide variety of compounds with different organoleptic properties, which will slightly evolve during bottle aging due to the limited quantities of oxygen penetrating through the closures [1,2,3,4]. The increase of dissolved oxygen (DO) in wines means the replenishment of oxygen is higher than the consumption of oxygen by antioxidants (such as phenolic compounds), while the decrease of DO represents a relatively higher consumption of oxygen [5]. Different types of closures exhibit different abilities in preventing oxygen penetration due to their structural differences [6,7]. Natural corks are the traditional choice of closure in the wine industry, but other types of closures are also used by wine producers. Agglomerated corks and technical corks are made of offcuts of oak wood, thus can be named oak-based corks as opposed to natural corks. In comparison with oak-based corks, polymer synthetic plugs and screw caps are more economical and less dependent on the raw material limitation.

Bottle aging is an important period when wine’s flavor characters must be preserved as much as possible. The main function of closures is to ensure a good seal and to prevent any organoleptic deterioration of wine during storage. However, during bottle aging, various reactions may occur, such as oxidation, hydrolysis, and reactions caused by charge transfer and formation of covalent bonds, which will influence wine flavor evolution [6]. Wine aroma quality like the fruity and floral perception usually decreases due to the diminishment of critical aroma compounds including long-chain aliphatic acid ethyl esters, terpenes, and norisoprenoids [8,9]. Wine astringency also decreases because of a decline in the mean polymerization degree of tannins [10], while the hue and color stability usually increase due to the formation of stable orange-yellow pigments such as pyranoanthocyanins [11]. So far, most research observing wine flavor changes during bottle aging has focused on dry red wines [1,12], or is only concerned about the flavor quality, determined either by volatile compounds or non-volatile compounds [3,4,13,14]; comprehensive investigations monitoring the evolution of rosé and dry white wines’ flavor profiles and oxidation patterns during bottle aging are quite limited.

The objectives of our research were: (1) understanding the evolution of flavor compounds of rosé and white wines during an 18-month bottle aging; (2) observing the oxidation pattern differences of rosé and white wines during an 18-month bottle aging and finding out the possible reasons that caused the differences; (3) comparing the effects of natural cork and its alternatives on the flavor profiles of rosé and white wines after an 18-month bottle aging.

## 2. Results and Discussion

### 2.1. Evolution of Phenolic Compounds and Dissolved Oxygen during Bottle Aging

**Figure 1:**
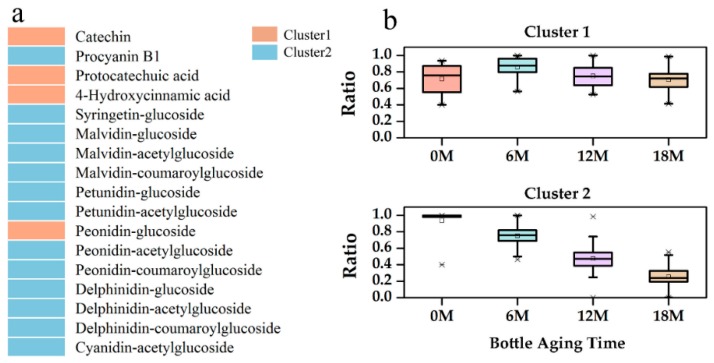
Statistical clustering for change trends of phenolic compounds in all rosé wines during an 18-month bottle aging. (**a**) General phenolic profile of rosé wines; (**b**) phenolic compounds’ change trends.

Seventeen phenolic compounds were quantified in rosé wines (Figure 1a) and only four non-anthocyanin compounds were quantified, which might be due to the short maceration time during winemaking. Phenolic compounds in all rosé wine samples were clustered into two groups using k-means algorithm after normalization process (dividing the concentration by the maximum value of each compounds among all samples). A boxplot based on the normalized data was carried out to exhibit the change trend of phenolic compounds of each cluster, the fluctuating group (Cluster 1) and the decreasing group (Cluster 2) (Figure 1b). The majority of phenolic compounds belonged to Cluster 2, which was similar to previous reports on red wines [11,15]. Considering the limited non-anthocyanin phenolics detected in rosé wines, the decrease of major anthocyanins should not be strongly correlated with the formation of polymeric pigments [10]. Therefore, these compounds should participate in reactions such as oxidation and degradation [16]. Only caffeic acid and 4-hydroxycinnamic acid were detected in white wines, which increased from 1.07 ± 0.17 mg/L to 1.98 ± 0.26 mg/L and 0.97 ± 0.04 mg/L to 1.6 ± 0.11 mg/L, respectively (see Table S1), due to hydrolysis of their tartaric esters [10].

**Figure 2:**
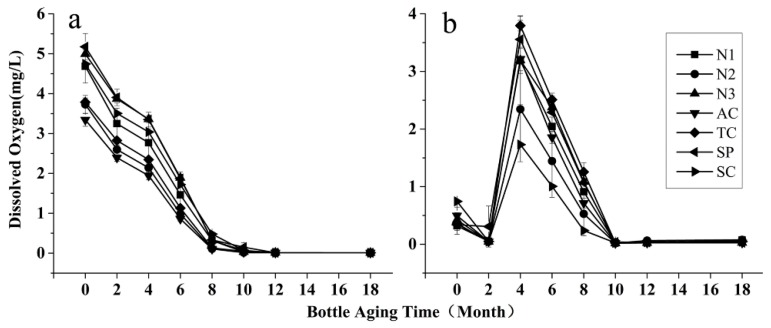
Change trends of DO in rosé (**a**) and white wines (**b**) during an 18-month bottle aging. (Symbols and abbreviations: N1: natural cork-1; N2: natural cork-2; N3: natural cork-3; AC: agglomerated cork; TC: ‘1+1′ technical cork; SP: polymer synthetic plug; SC: screw cap.)

The level of dissolved oxygen (DO) in wines mainly depends on oxygen concentration in the headspace at bottling and the ingress rate of oxygen into the bottle through closures [9]. During bottling, wines are exposed to air and thus have a chance to absorb oxygen. The oxidation substrates, generally phenolic compounds in wines, are positively correlated to wines′ oxygen absorption capacity [6]. Rosé and white wines have unique DO evolution behaviors (Figure 2). In all rosé wines, the concentrations of DO decreased constantly during the first 10 months of bottle aging (Figure 2a), possibly because their major antioxidants, the phenolic compounds, were decreasing due to oxidation (Figure 1). However, in all white wines, the change trends of DO showed a slight decrease in the first two months, and then a drastic fluctuation (Figure 2b). The accumulation of DO meant the replenishment of oxygen was higher than the consumption. The oxygen dissolved in white wines probably came from the headspace oxygen, which was much higher in white wines (5.12 ± 0.76 mg/L) than in rosé wines (1.57 ± 0.73 mg/L). Moreover, caffeic acid and 4-hydroxycinnamic acid in white wines are good antioxidants. However, they might react slowly or not react at all until their concentrations reach a certain degree due to the hydrolysis of their tartaric esters, just like the formation of pinotins, a group of pyranoanthocyanins slowly formed by reactions between hydroxycinnamic acids and anthocyanins during wine aging [17]. After a four-month bottle aging, the accumulation of caffeic acid and 4-hydroxycinnamic acid was sufficient to trigger oxidation reactions, leading to a drastic decrease in DO (Figure 2b). Furthermore, white wines sealed with a screw cap had the lowest concentration of DO during bottle aging, which means a screw cap might provide better preservation for white wines. The DO concentrations were below the detection limit after 10 months of bottle aging for all rosé and white wines, indicating that the oxygen replenished from outside of the closures was far less than the oxygen consumption potential of wines. According to the results, we believe that although closures act as an oxygen barrier [18], DO’s change trends during preservation depend more on the wine type and also the initial oxygen dissolved during the bottling process.

### 2.2. Evolution of Volatile Compounds during Bottle Aging

**Figure 3:**
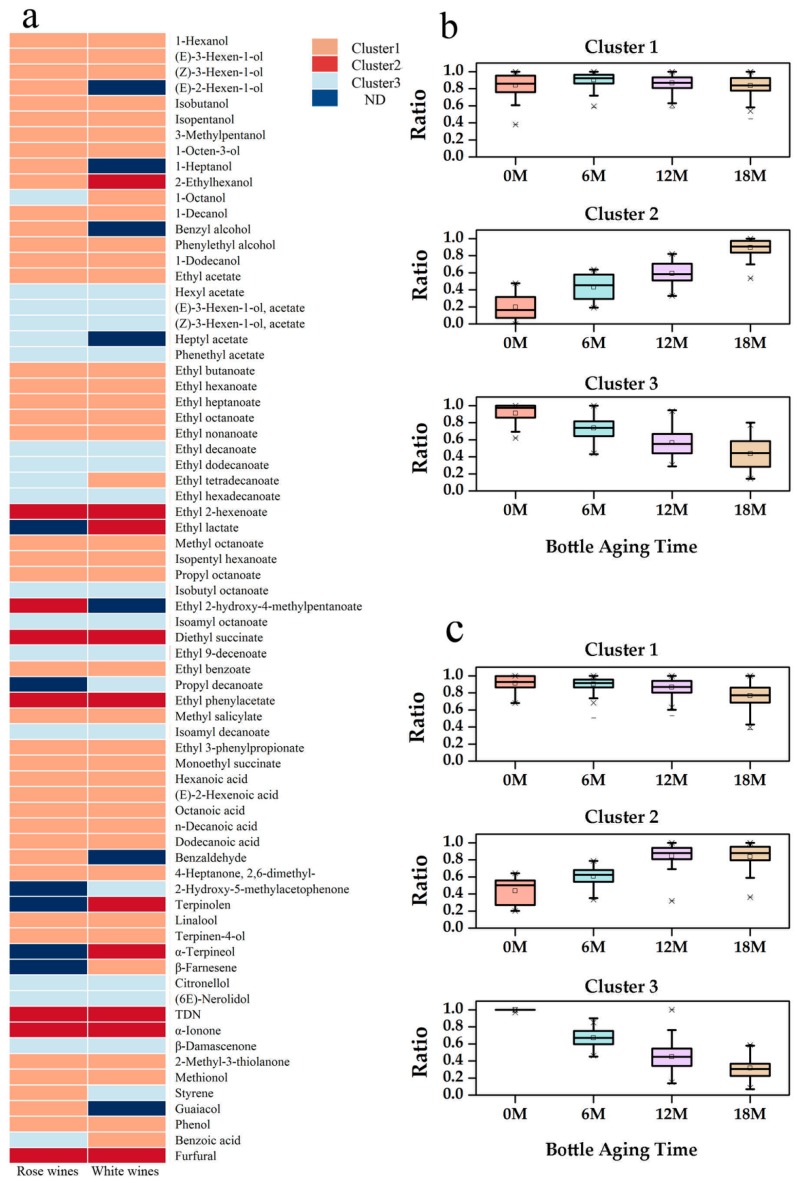
Change trends of volatile compounds during an 18-month bottle aging. (**a**) General volatile profile of rosé and white wines; (**b**) volatile compounds’ change trends in rosé wines; (**c**) volatile compounds’ change trends in white wines.

**Figure 4:**
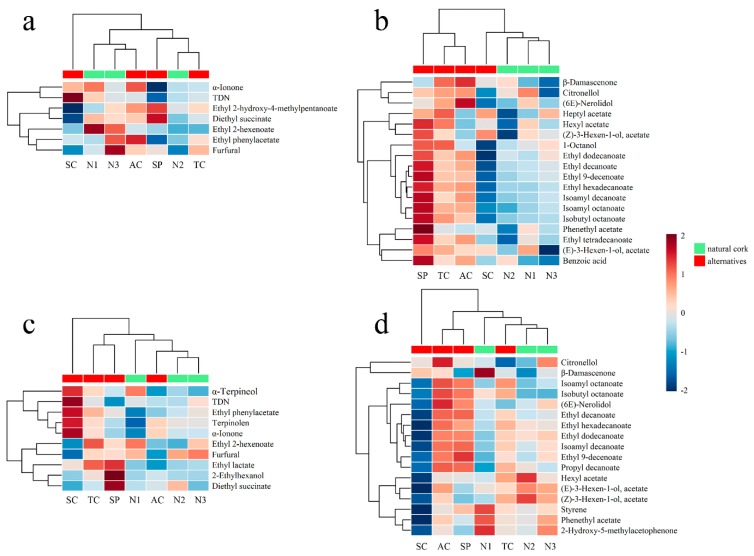
Clustering analysis of wines sealed with natural corks and their alternatives based on absolute values of the differences between final concentrations and initial concentrations. (**a**) Difference value of increasing volatile compounds in rosé wines; (**b**) difference value of decreasing volatile compounds in rosé wines; (**c**) difference value of increasing volatile compounds in white wines; (**d**) difference value of decreasing volatile compounds in white wines.

There were 72 volatile compounds quantified in this study, including 15 alcohols, 32 esters, five aliphatic acids, seven terpenes, three norisoprenoids and 10 other volatile compounds (Figure 3a). Detailed information is shown in Table S2. These two types of wines shared similar aromatic profiles, except there were more terpenes only quantified in whites (terpinolene, α-terpineol, and β-farnesene) to represent floral notes (Figure 3a). (E)-2-hexen-1-ol, 1-heptanol, benzyl alcohol, heptyl acetate, ethyl 2-hydroxy-4-methylpentanoate, benzaldehyde, and guaiacol were only quantified in rosé wines. Some volatile compounds, such as ethyl decanoate, n-decanoic acid, and dodecanoic acid, were reported to be associated with the oxidation of wines [8]. Wine oxidation was responsible for some unpleasant sensory descriptors (‘green apple,’ ‘cooked potato,’ and ‘curry’), and could also interact with remaining pleasant aroma compounds, leading to the suppression of certain positive attributes [19]. The evolution of volatile compounds in wines during bottle aging was analyzed by k-means algorithm. It was clear that all compounds could be classified into three groups, namely, Cluster 1, Cluster 2, and Cluster 3, shown in the boxplot based on the normalized data from k-means algorithm (Figure 3b,c). Cluster 1 compounds stayed stable and were mostly alcohols, some ethyl esters, aliphatic acids, and a few terpenes (linalool and terpinen-4-ol). Compounds in Cluster 2 and 3 exhibited an increasing and decreasing trend, respectively, which indicated that they were the main changing compounds. Overall, more volatile compounds had a decreasing trend than an increasing trend during bottle aging. Almost half of the esters detected in our study were found in Cluster 3, which corroborates previous studies’ finding that esters decreased during ageing due to hydrolysis [2,20]. By calculating the absolute values of the differences between final concentrations (after an 18-month bottle aging) and initial concentrations of those changing compounds, we used clustering analysis to differentiate wines sealed with natural corks and their alternatives (Figure 4).

For rosé wines, wines treated with similar closures (natural corks or alternatives of natural corks) did not show much consistency based on Cluster 2 compounds (Figure 4a), while Cluster 3 compounds could precisely distinguish wines with natural corks from their alternatives (Figure 4b). In Figure 4b, the difference between final concentration and initial concentration of Cluster 3 compounds showed less of a heat response in rosé wines sealed with natural corks than those sealed with alternative closures, which meant natural corks could better prevent aroma loss, especially for acetates (heptyl acetate, hexyl acetate, 3-hexen-1-ol, phenethyl acetate). β-Damascenone and (6E)-nerolidol decreased more in rosé wines sealed with a ‘1+1′ technical cork and agglomerated cork, while the compounds in wines with a polymer synthetic plug seemed to have the largest decrease rate during bottle aging.

As for white wines, agglomerated cork and ‘1+1′ technical cork were mixed with natural corks in a clustering analysis for compounds in Cluster 2 and 3, mainly because they were all oak-based closures (Figure 4c,d). In Cluster 2, α-ionone and TDN had a relatively lower increase rate in wines with natural corks compared to their alternatives, which might be due to the ‘scalping phenomenon’ of corks. Some studies have showed that cork and synthetic closures may scalp several aroma compounds from wine, such as TDN and methoxypyrazines [18] (Figure 4c). In Figure 4d, white wines with a screw cap had the most compounds that exhibited less heat response among all samples, which meant the screw cap was better ay preserving aroma quality in white wines. This might be due to there being the lowest level of DO concentration in white wines sealed with a screw cap (Figure 2b). It was those long-chain aliphatic acid esters that decreased less in both rosé and white wines sealed with a screw cap, such as ethyl decanoate, ethyl 9-decenoate, ethyl dodecanoate, ethyl hexadecanoate, isoamyl decanoate, isoamyl octanoate, and isobutyl octanoate, which could differentiate a screw cap from other closures. The result corresponded to a previous study that found that long-chain aliphatic acid esters would decrease with a higher oxidation level [8], while a screw cap seemed to be the best choice in terms of minimizing wine oxidation [1,9]. On the contrary, agglomerated cork and polymer synthetic plug could lead to more loss in the aroma compounds (Figure 4d).

### 2.3. Flavor Profile Analysis of Wines after an 18-Month Bottle Aging

**Figure 5:**
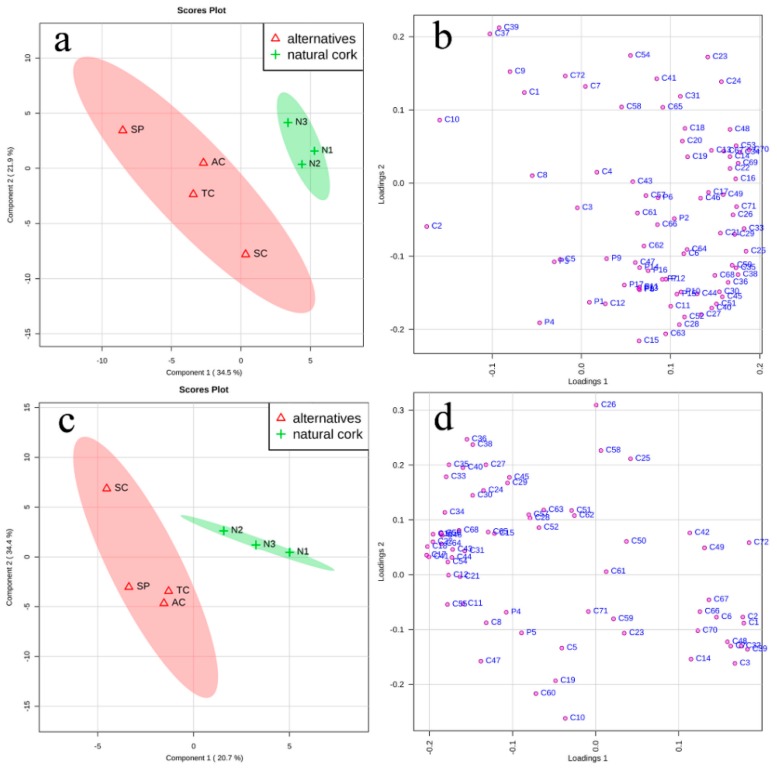
PLS-DA of flavor compounds in 18-month bottle-aged wines treated with natural corks and their alternatives. (**a**) PLS-DA model for rosé wine differentiation; (**b**) scattering plot of PLS-DA model for rosé wines; (**c**) PLS-DA model for white wine differentiation; (**d**) scattering plot of PLS-DA model for white wines. Note: Flavor compounds′ numbers in (**b**,**d**) are provided in Tables S1,S2.

During bottle aging, wines are expected to maintain their original organoleptic characters as much as possible. In this section, partial least squares-discriminate analysis (PLS-DA) of different flavor compounds (phenolic and volatile compounds) was conducted in wines after an 18-month bottle aging. Wines sealed with three natural corks (N1, N2, N3) were set up as a group, while wines sealed with other closures (AC, TC, SP, SC) were set up as a single group of natural cork alternatives to see the effect of natural corks and their alternatives on wines’ flavor profile (Figure 5). The use and interpretation of the VIP (variable importance in projection) values obtained from the PLS-DA model make it possible to determine potential flavor markers in the classes selected. Besides calculating the VIP values in PLS-DA, a one-way ANOVA test was also applied to check the statistical differences between different flavor compounds and guarantee a more statistically reliable selection of critical flavor markers (Table 1). Moreover, by calculating OAVs and chromatic parameters in all initial wines (prior to bottle aging) and final wines (after an 18-month bottle aging), the aromatic and chromatic characters of these wines were also compared.

#### 2.3.1. Flavor Compounds

In rosé wines, principal component 1 (PC1) and principal component 2 (PC2) accounted for 56.4% of the total variance (Figure 5a). According to variable importance in projection values (VIP > 1) of the first two PCs from the PLS-DA model and *P* values (*p* < 0.05) from a one-way ANOVA test, differentiated flavor compounds in rosé wines sealed with natural corks and their alternatives were selected (Table 1). Closures seemed to have more effect on volatile compounds than phenolic compounds because those differentiated flavor compounds were all volatile compounds. Only rosé wines with natural corks were in the quadrant where PC1 and PC2 were both positive. PC1, which accounted for 34.5% of the total variance, could distinguish wines sealed with natural corks from those with a polymer synthetic plug (SP) (Figure 5a). As shown in Figure 5b, the positive direction of PC1 was mostly driven by esters, such as ethyl octanoate (C25), methyl octanoate (C33), isopentyl hexanoate (C34), and isoamyl octanoate (C38). Except for 2-ethylhexanol (C10), the other differentiated compounds all lay in the positive part in PC1. Ethyl heptanoate (C24), hexanoic acid (C48), β-damascenone (C65), and guaiacol (C69) had a higher concentration in wines with natural corks (see Table S2). All phenolic compounds, especially 4-hydroxycinnamic acid (P4) and catechin (P1), drove PC2 in the negative direction, where wines with a screw cap lay.

As for white wines, PC1 and PC2 accounted for 55.1% of the total variance (Figure 5c). Since phenolic compounds are quite limited in white wines, aroma properties play a more important role from an organoleptic perspective. According to the loading plot, the quadrant where PC2 was positive and PC1 was negative contained the most types of volatile compounds (alcohols, esters, aliphatic acids, terpenes, and norisoprenoids) (Figure 5d). Wines sealed with a screw cap lay in this quadrant. The most differentiated compounds (VIP > 1, *P* < 0.05) in white wines were 2-ethylhexanol (C10), 1-decanol (C12), 2-hydroxy-5-methylacetophenone (C55), β-farnesene (C60), α-ionone (C64), and styrene (C68). Those volatile compounds characterized the negative direction of PC1 (Table 1, Figure 5d) to distinguish wines sealed with a screw cap from those with natural corks. Wines sealed with agglomerated cork (AC), ‘1+1′ technical cork (TC), and polymer synthetic plug (SP) were together in the third quadrant, close to the ‘zero’ of plot score.

Comparing the flavor compounds in rosé and white wines, wines with natural corks could be distinguished from their alternatives by the results of PLS-DA. The only differentiated flavor compound rosé and white wines had in common was 2-ethylhexanol, which was related to the citrus odor. The quadrant where rosé wines with natural corks lay contained more compounds, indicating that natural corks could perform better in preventing flavor compounds from loss during bottle aging in rosé wines. Whereas, in white wines, it was wines with a screw cap that stood out with more volatile compounds, which might be due to having the best sealing environment. This result was also in accordance with a previous study in Semillon wines that bottling with a screw cap tends to lead to a higher fruity, citrus sensory score and less of an oxidized aroma [9].

#### 2.3.2. Flavor Characters

To learn more about the aroma profile in rosé and white wines, Duncan’s multiple range tests based on OAVs were applied to identify which types of closures had a different effect on wines after an 18-month bottle aging (Table 2). Overall, the rosé and white wines were both fruity, floral, and sweet in odor, which makes sense given that most of the volatile compounds quantified in this study were esters and the thresholds of terpenes and norisoprenoids were usually low. White wines exhibited more ‘berry’ and ‘sweet’ odors, mainly due to the high level of ethyl esters, such as ethyl acetate, ethyl octanoate, ethyl decanoate, and ethyl dodecanoate (Table S2). During an 18-month bottle aging, the total intensity of aroma in both types of wines declined prominently, especially ‘tropical fruity,’ ‘floral,’ ‘berry,’ and ‘sweet’ aroma descriptions. The results indicated that the period of bottle aging was likely to contribute to the loss of aroma properties. The decrease was more obvious in white wines, as the studies showed that white wines were very unstable and lost their desirable fresh and fruity characters over time [20]. According to the results of Duncan’s multiple range tests, the difference between different closures mainly concerned the ‘berry’ and ‘sweet’ odors. Ethyl acetate, ethyl 2-hydroxy-4-methylpentanoate, ethyl decanoate, and β-damascenone were representative compounds of those odorant series. Wine samples sealed with a screw cap contained a higher concentration of long-chain aliphatic acid esters, which might be the reason why it performed better in preventing ‘berry’ and ‘sweet’ odors from declining. There was no significant difference between rosé wines sealed with three natural corks (N1, N2, N3) and a screw cap (SC), except for the ‘berry’ aroma description. On the contrary, OAVs in rosé wines sealed with a polymer synthetic plug (SP) were significantly lower than those sealed with other closures after an 18-month bottle aging. White wines with agglomerated cork (AC) and polymer synthetic plug (SP) had the lowest OAVs among other types of closures, indicating that those two types of closures were not appropriate for the preservation of white wines during bottle aging.

**Figure 6:**
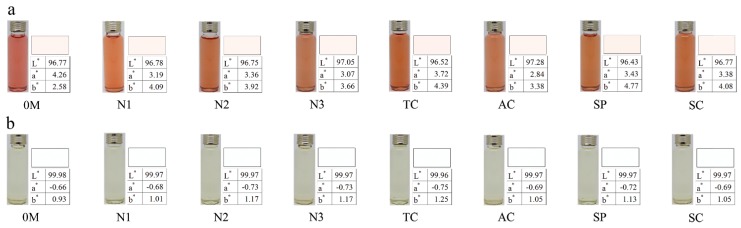
Sample photos and chromatic parameters of rosé (**a**) and white wines (**b**) prior to and after an 18-month bottle aging.

As for chromatic characters, due to the decrease of anthocyanins in rosé wines during bottle aging, a^*^ values were much lower, while b^*^ values were higher in final wines than in initial wines (Figure 6a), indicating that the hue tended to undergo a yellowing phenomenon after 18 months of bottle aging. Although the ΔE values of rosé wines in our research ranged from 1.62 to 2.37 and the chromatic difference in wines was perceivable by human eyes when the ΔE value was above 2.8 [28], judging from the direct comparison in Figure 6a, visual differences existed in all rosé wines after an 18-month bottle aging compared to the initial wines. This may be due to the extreme brightness caused by low color substances in rosé wines, since it was reported that an ΔE value >1.0 in model solutions was visually perceivable by the human eye [28]. The ΔE value of white wines ranged from 0.08 to 0.33, corresponding to no visual differences in all white wines after an 18-month bottle aging (Figure 6b).

## 3. Materials and Methods

### 3.1. Wine Samples

‘Cabernet Sauvignon’ rosé wines and ‘Chardonnay’ dry white wines were made strictly according to the local published winemaking standards in October 2014, at Shandong Taila Winery Co., Ltd. (Rushan, Shandong, China). Detailed information about the two original wine samples is given in Table S3. Bottling was performed at the packaging line of this winery to guarantee a 750 mL volume of each bottle. Seven types of closures were used for treatment, including three different natural corks [named natural cork-1 (N1), natural cork-2 (N2), and natural cork-3 (N3)] and four natural cork alternatives [‘1+1′ technical cork (TC), agglomerated cork (AC), polymer synthetic plug (SP) and screw cap (SC)]. The physical indexes of these closures are provided in Table S4. For each closure treatment, 30 bottles of wine were collected as samples. After bottling, wine samples were stored in a cellar, with an average temperature of 16 ± 1 °C and a relative humidity of 65 ± 5%. The length of the experiment was 18 months.

### 3.2. Oxygen Measurements

The dissolved oxygen (DO) levels were recorded by noninvasive oxygen sensors (5 mm sensor spots PSt3, NomaSense O_2_ P6000, Yantai Vinventions Co., Ltd., Yantai, China). For each closure treatment, three bottles of wine were selected randomly to equip one sensor positioned at mid height and one in the neck of the bottle. Wine samples were analyzed every two months for oxygen data acquisition.

### 3.3. Flavor Compounds Detection

Phenolic compounds were detected via high-performance liquid chromatography/triple-quadrupole tandem mass spectrometry (HPLC-QqQ-MS/MS) method using an Agilent series 1200 instrument fitted with a Poroshell 120 EC-C18 column (150 × 2.1 mm, 2.7 μm, Agilent Technologies, Santa Clara, CA, USA). The method was established and validated in our earlier work, and is reliable for detecting and quantifying 45 non-anthocyanin and 95 anthocyanin compounds [29,30]. Volatile compounds were detected using headspace solid phase micro-extraction (HS-SPME) with a 2 cm DVB/CAR/PDMS 50/30 µm SPME fiber (Supelco, Bellefonte, PA, USA) and an Agilent 7890 gas chromatography kit equipped with an Agilent 5975 mass spectrometer (GC–MS). The details were given in our previous study [31]. Retention indices (RI) [adjected by C7–C24 n-alkane series (Supelco)] were compared with those in the NIST11 database via an Automatic Mass Spectral Deconvolution & Identification System (AMDIS) for identification. The ChemStation software (Agilent Technologies, Inc.) was used to calculated the peak areas and quantification was conducted on the basis of the calibration curve of volatile compound standards purchased from Sigma-Aldrich (St. Louis, MO, USA).

Three bottles of wine samples from each closure treatment were collected randomly every six months for flavor compound detection. Each sample was analyzed in duplicate.

### 3.4. Flavor Analysis

By calculating the ratio of the volatile compound concentration to its odor perception threshold, the odor activity value (OAV) was obtained. This analysis was used to assess the potential contribution of individual volatile compounds to wine aroma.

Chromatic parameters were measured in triplicate using a spectrophotometer Shimadzu UV-Vis 2600 (Shimadzu, Kyoto, Japan) [30] and analyzed via CIELab system to detect the lightness (L^*^), reddish attribute (a^*^), and yellowish attribute (b^*^) of each wine [32]. The visual change in wine, described as ΔE, was calculated using the following equation [28]:

ΔE* = [(ΔL^*^)^2^ + (Δa^*^)^2^ + (Δb^*^)^2^]^1/2^ (1)

### 3.5. Statistical Analysis

K-means algorithm and clustering analysis were conducted respectively by ‘kmeans’ function and ‘pheatmap’ function in R environment (3.4.0) (http://www.r-project.org/). Partial least squares-discriminate analysis (PLS-DA) was conducted by MetaboAnalyst 4.0 (http://www.metaboanalyst.ca/). One-way ANOVA tests and Duncan’s multiple range tests were carried out using SPSS software version 20.0 for Windows (SPSS Inc., Chicago IL, USA).

## 4. Conclusions

In conclusion, by assessing the dissolved oxygen (DO), phenolic and volatile compounds, OAV and chromatic parameters, a comprehensive understanding of the evolution of oxidation patterns and flavor profiles in rosé and dry white wines during an 18-month bottle aging was established. Those two types of wines had different change trends of DO because of their unique phenolic compositions and the initial concentration of headspace oxygen, which makes it important for winemakers to control the bottling process. Many volatile compounds exhibited a decreasing trend during bottle aging and the total intensity of aroma declined obviously compared with their counterparts prior to bottling in all wines. Rosé wines sealed with natural corks and screw caps exhibited no significant difference after an 18-month bottle aging, except for in the ‘berry’ aroma description, while a polymer synthetic plug could lead to a greater loss of aroma quality in rosé wines during bottle aging. Agglomerated corks and polymer synthetic plugs were not appropriate for the preservation of white wines, while screw caps behaved better than any other closures in maintaining aroma compounds in white wines. After an 18-month bottle aging, rosé wines exhibit a difference in color, whereas this phenomenon was not observed in white wines. Our study has extended the research into the evolution of flavor profiles in rosé and white wines; further attention should be given to the wines’ flavor chemistry and quality control during bottle aging.

## Figures and Tables

**Table 1:** Differentiated flavor compounds in rosé and white wines (VIP > 1, *p* < 0.05).

No. ^a^	CAS	Compounds ^b^	‘Cabernet Sauvignon’ Rosé Wines			‘Chardonnay’ Dry White Wines		
			VIP1	VIP2	*p* values	VIP1	VIP2	*p* values
C10	104-76-7	2-Ethylhexanol	1.145	1.055	**0.024**	1.045	1.229	**7.07 × 10^−7^**
C12	112-30-1	1-Decanol	0.286	0.617	0.001	1.443	1.022	**0.035**
C24	106-30-9	Ethyl heptanoate	1.876	1.720	**0.022**	0.348	1.025	0.992
C29	124-06-1	Ethyl tetradecanoate	1.404	1.245	**0.022**	0.155	0.944	0.029
C33	111-11-5	Methyl octanoate	1.266	1.187	**0.021**	0.711	1.121	0.954
C38	2035-99-6	Isoamyl octanoate *	1.136	1.111	**0.002**	0.117	1.44	0.678
C48	142-62-1	Hexanoic acid **	1.655	1.464	**0.022**	0.717	0.917	0.975
C55	1450-72-2	2-Hydroxy-5-methylacetophenone	Trace	Trace	Trace	1.673	1.218	**0.013**
C60	18794-84-8	β-Farnesene	Trace	Trace	Trace	1.053	0.963	**0.000**
C64	8013-90-9	α-Ionone *	0.341	0.851	0.003	1.464	1.039	**0.037**
C65	23726-93-4	β-Damascenone **	1.301	1.274	**0.014**	0.428	0.883	0.749
C68	100-42-5	Styrene	0.691	0.934	0.001	1.121	0.847	**0.010**
C69	90-05-1	Guaiacol *	1.777	1.575	**0.04**	Trace	Trace	Trace

The data in bold were the most differentiated compounds (VIP>1, *p* < 0.05); ^a.^ The No. was in accordance with Table S2; ^b.^ * OAV > 0.1, ** OAV > 1.0.

**Table 2:** OAVs in all initial wines (0M) and final wines (after an 18-month bottle aging).

**Aroma Description**	**0M**	**N1**	**N2**	**N3**	**TC**	**AC**	**SP**	**SC**
	**‘Cabernet Sauvignon’ rosé wines**							
Tropical Fruity	56.01 ± 5.02	33.11 ± 1.07ab	31.9 ± 1.13ab	33.68 ± 2.62a	29.96 ± 0.65b	30.51 ± 0.11ab	30.26 ± 0.67b	31.77 ± 1.49ab
Floral	35.17 ± 3.08	12.98 ± 0.06ab	12.18 ± 0.5cd	13.48 ± 0.2a	11.49 ± 0.08de	11.28 ± 0.32e	12.44 ± 0.2bc	12.34 ± 0.37bc
Berry	14.86 ± 1.33	10.95 ± 0.65b	11.15 ± 0.8b	10.68 ± 1.08bc	9.74 ± 0.37bc	9.34 ± 0.21c	7.51 ± 0.2d	13.57 ± 0.62a
Herbaceous/Vegetal	1.27 ± 0.07	1.27 ± 0.05ab	1.31 ± 0.06a	1.27 ± 0.08ab	1.24 ± 0.03ab	1.24 ± 0.01ab	1.18 ± 0.02b	1.3 ± 0.06a
Chemical	8.57 ± 0.8	8.94 ± 0.43ab	8.47 ± 0.31ab	8.69 ± 0.94ab	7.83 ± 0.2b	8.09 ± 0.19b	6.65 ± 0.18c	9.32 ± 0.5a
Fatty	9.66 ± 0.27	9.01 ± 0.32a	9.04 ± 0.58a	9.01 ± 0.36a	8.8 ± 0.14a	8.58 ± 0.16a	7.24 ± 0.32b	8.99 ± 0.19a
Sweet	26.05 ± 2.21	21.02 ± 1.18ab	21.14 ± 1.16ab	20.97 ± 2.41ab	19.07 ± 0.65bc	19.15 ± 0.45bc	17.02 ± 0.43c	23.01 ± 1.12a
	**‘Chardonnay’ dry white wines**							
Tropical Fruity	44.8 ± 1.19	28.13 ± 0.21a	26.77 ± 1.12a	28.44 ± 1.13a	26.62 ± 2.71a	27.02 ± 0.5a	27.86 ± 2.97a	28.61 ± 1.03a
Floral	19.81 ± 0.35	8.93 ± 0.21b	9.66 ± 0.04a	9.41 ± 0.11a	9.47 ± 0.27a	9.43 ± 0a	9.6 ± 0.24a	9.57 ± 0.17a
Berry	21.12 ± 0.49	8.73 ± 0.39ab	8.13 ± 0.12bc	8.11 ± 0.61bc	7.56 ± 0.65bc	6.66 ± 0.42c	6.72 ± 1.16c	10.16 ± 0.54a
Herbaceous/Vegetal	1.3 ± 0.02	1.01 ± 0.17a	0.89 ± 0.03a	0.88 ± 0.04a	0.85 ± 0.04a	0.83 ± 0.02a	0.88 ± 0.1a	0.93 ± 0.05a
Chemical	10.68 ± 0.4	6.43 ± 0.02ab	5.99 ± 0.3ab	6.43 ± 0.52ab	5.82 ± 0.73b	5.68 ± 0.3b	5.33 ± 0.95b	7.14 ± 0.32a
Fatty	12.39 ± 0.36	11.74 ± 0.62ab	11.45 ± 0.48ab	10.77 ± 0.16b	11.46 ± 0.61ab	11.14 ± 0.34b	10.4 ± 0.74b	12.9 ± 0.94a
Sweet	34.94 ± 0.81	20.58 ± 0.15ab	18.68 ± 0.94ab	20.01 ± 1.09ab	18.34 ± 2.24ab	17.87 ± 0.59b	18.58 ± 2.87ab	22.09 ± 1.11a

Odor activity values (OAVs) were shown through average ± standard error. The odor thresholds were taken from [21,22,23,24,25,26,27]. The details are shown in Table S2. Different letters in the same row indicate significant differences at *p* < 0.05 by Duncan’s multiple-range test.

## Supplementary Materials

Supplementary Materials are available online. Table S1: Phenolic compounds identified in this work; Table S2: Volatile compounds identified in this work and their aroma parameters; Table S3: Detailed information of two original wine samples; Table S4: Physical index of various bottle closures used in this work.
